# Quality of life, fatigue and mental health in patients with the m.3243A > G mutation and its correlates with genetic characteristics and disease manifestation

**DOI:** 10.1186/s13023-016-0403-5

**Published:** 2016-03-18

**Authors:** Christianne Verhaak, Paul de Laat, Saskia Koene, Marijke Tibosch, Richard Rodenburg, Imelda de Groot, Hans Knoop, Mirian Janssen, Jan Smeitink

**Affiliations:** Department of Medical Psychology, Radboud University Nijmegen Medical Center, Nijmegen, The Netherlands; Department of Pediatrics, Radboud Center for Mitochondrial Medicine, Radboud University Nijmegen Medical Center, Nijmegen, The Netherlands; Department of rehabilitation, Radboud Center for Mitochondrial Medicine, Radboud University Nijmegen Medical Center, Nijmegen, The Netherlands; Department of Internal Medicine; Radboud Center for Mitochondrial Medicine, Radboud University Nijmegen Medical Center, Nijmegen, The Netherlands

**Keywords:** Mitochondrial disease, m.3243A > G mutation, Quality of life, Patient reported outcomes, Fatigue, Mental health, Participation

## Abstract

**Background:**

Mitochondrial disorders belong to the most prevalent inherited metabolic diseases with the m.3243A > G mutation reflecting being one of the most common mutations in mitochondrial DNA. Previous studies showed little relationship between mitochondrial genetics and disease manifestation. Relationship between genotype and disease manifestation with patient reported quality of life and other patient reported outcomes is still unexplored.

**Methods:**

Seventy-two out of the 122 invited adult patients with m.3243A > G mutation completed online standardized questionnaires on quality of life, functional impairment, fatigue and mental health as assessed by the RAND-SF36, the Sickness Impact Profile (SIP), the Checklist Individual Strength (CIS) and the Hospital Anxiety and Depression scale (HADS). Data were related to clinical manifestation reflected by the Newcastle Mitochondrial Disease Adult Scale (NMDAS) score and heteroplasmy levels of the mutation in urine epithelial cells.

**Results:**

Patients reported impaired quality of life. Sixty percent showed severe levels of fatigue, and 37 % showed clinical relevant mental health problems, which was significantly more than healthy norms. These patient reported health outcomes showed negligible relationship with levels of heteroplasmy (*r* = <.30) and weak (.30 < r < .50) to moderate (.50 < r < .70) relationship with clinical manifestation.

**Conclusions:**

Patient reported outcomes on quality of life, fatigue and mental health problems, are only partly reflected by clinical assessments. In order to support patients more effectively, integration of patient reported outcomes, alongside symptoms of their disease, in clinical practice is warranted.

## Background

Mitochondrial disorders, as a group, belong to the most prevalent inherited metabolic diseases. The incidence of congenital mitochondrial disorders based on oxidative phosphorylation (OXPHOS) defects is at least 1:8500 of all life births [1]. Mitochondrial diseases can result from mutations in either nuclear or mitochondrial DNA and show great variability in expression. Although new pharmacological interventions are being explored in cells and animals, currently no cure or substantially alleviating therapy is available for these disorders and care is focused on alleviating the broad range of symptoms of the disease. Mitochondrial diseases have a complex and heterogeneous expression with often more than one system structurally and/or functionally affected (e.g. brain, muscles, the heart). Previous studies showed little relationship between mitochondrial genetics and clinical manifestation [2–5] (Chinnery et al. 1997; Grady et al. 2014; De Laat et al. 2012; Koene et al. 2013). In addition, a recent study that took patient reported outcomes on fatigue into account reported little relationship between these outcomes and genotype and clinical manifestation either [6] (Gorman et al. 2015). Information about patients’ self reported quality of life and impact of the disease in their daily life is important to be able to focus care on patients’ most important complaints, as well as to further understand the relationship of biological and clinical parameters with these patient reported outcomes.

In a study of 78 parents of children with a mitochondrial disorder, Koene et al. showed in 2013 that the most burdensome complaints of patients included fatigue, problem behavior, muscle weakness and a high degree of limitations in daily activities [5] (Koene et al., 2013). Recently, Gorman et al. [6] (2015) supported the importance of fatigue in a group of patients with a variety of mitochondrial disorders.

This is in line with studies in patients with other chronic conditions such as T1DM [7], multiple sclerosis [8], and Ehlors-Danlos syndrome [9] showing fatigue as an important burden. These studies too, show little relationship of patient reported outcomes such as fatigue and quality of life with clinical manifestation. The aim of the present study is to investigate patient reported outcomes in terms of quality of life, functional impairment, fatigue and mental health. In addition, this study is aimed to assess the relationship between these patient reported outcomes and disease manifestation as well as genotype.

We focused on a group of patients with the m.3243A > G mutation reflecting one of the most common genetic causes for mitochondrial disorders, often referred to as acronym Mitochondrial Encephalomyopathy Lactic Acidosis and Stroke like episodes (MELAS) [10] (Pavlakis, Phillips, DiMauro, De Vivo, & Rowland, 1984) and/or MIDD (maternally Inherited Diabetes and Deafness). The disease spectrum of patients with the m.3243A > G mutation is multidimensional. Patients suffer from various mainly neurological problems (such as stroke like episodes, epilepsy, dementia, migraine, muscle pain or psychiatric problems), but also from a broad range of other problems importantly affecting their daily functioning.

## Methods

### Sample

All 122 patients, age 18 or above, with a mitochondrial disease due to the m.3243A > G mutation in leucocytes, urinary epithelial cells and buccal mucosa, under care of the Nijmegen Center for Mitochondrial Disorders at the Radboud University Medical Centre, were invited to participate in this longitudinal observational study.

### Procedure

After given informed consent, patients received an email with a link to a private, secure website that presented a set of questionnaires that could be administered at home, at time of first access or later. Patients were asked to complete the total set of questionnaires within one week. Assessments were repeated after two weeks (T2) and three months (T3). Newcastle Mitochondrial Disorder Assessment Scale [11] (Schaefer et al. 2006) was assessed during clinical consultations.

### Ethical committee

This study was approved by the local ethical committee of the Radboud University Medical Centre, Nijmegen, the Netherlands. All participants gave informed consent*.*

### Instruments

*Genotype* was assessed in terms of heteroplasmy levels in leucocytes, urine epithelial cells and in buccal mucosa. Genotyping procedure has been performed as described earlier [3] (De Laat et al. 2012).

*Disease Manifestation* was assessed by the Newcastle Mitochondrial Disease Adult Scale [11] (NMDAS; Schaefer et al. 2006). The NMDAS is a measure to monitor the clinical expression of the disease and consists of the following three sections: (1) Current functioning: general physical functioning of the patient in the past four weeks (2) System specific involvement to gain insight in the functioning of individual organ-systems. (3) Current clinical assessment gives insight in the current clinical status of the patient. Details of assessment as well as inter rater reliability are described previously [3] (De Laat et al. 2012).

We defined NMDAS scores of 1 to 5 as mild clinical manifestation, scores 6 to 20 as moderate and scores above 20 as severe clinical manifestation.

*Patient reported health outcomes* were assessed by online administered self-report on quality of life, functional impairment, fatigue and mental health.

*Quality of life* was assessed with the RAND-36. The RAND-36 assesses 7 dimensions of quality of life (physical functioning, social functioning, emotional functioning, general health status, perceived change in health status, sleep problems and pain). Scores on different scales range from 0 (maximum limitations) to 100 (optimal functioning). As reference group we used a Dutch norm group for general population of men and women age 18 to 75 years [12] (Van der Zee & Sanderman, 2012).

*Functional impairment* was assessed with the Sickness Impact Profile (SIP; [13, 14]). The SIP, is aimed to assess changes of conduct in everyday activities due to sickness and assesses the following dimensions of functioning: Sleep/rest, home management, mobility, social interaction, ambulation, alertness and intellectual functioning, work and recreation and pastime. Total scores ranged from 0 to 5799 with higher scores reflecting more impairment. Dutch norms are not available but scores of comparison groups with same age and sex were used consisting of 90 health controls in a study with 94 patients with Multiple Sclerosis [15] (Servaes et al. 2002).

*Perceived fatigue severity* was assessed with the fatigue severity subscale of the Checklist Individual Strength [16] (CIS; Vercoulen et al. 1999). The CIS is a questionnaire with 20 items that can be scored on a seven-point Likert scale. The CIS is designed to assess different dimensions of fatigue: fatigue severity (8 items scores ranging from 8 to 56), concentration (5 items scores ranging from 5 to 35), motivation (4 items scores ranging from 4 to 28) and physical activity (3 items, scores ranging from 3 to 21). Higher scores indicate higher levels of fatigue, more concentration problems, less motivation and low levels of physical activity. Studies indicated that the fatigue severity subscale was best indicator of patient reported fatigue related complaints. It consists of items like I feel fit, I feel tired, I feel powerless, I am rested, to complete on a 7-point Likert scale. A CIS-fatigue score of 35 or more was used to identify severe fatigue in correspondence with other studies [9, 15] (e.g. Servaes et al. 2002, Voermans et al. 2010). The CIS has good reliability and is used to assess perceived fatigue in several groups of patients with various medical conditions.

*Mental functioning* was assessed with the Hospital Anxiety and Depression scale [17, 18] (Dutch translation: Spinhoven et al. 1997), measuring symptoms of anxiety and depression. The HADS is specially designed to assess symptoms of depression in people with medical conditions by controlling for vital aspects of depression that could easily interfere with symptoms of the disease and showed good reliability and validity.

### Statistics

Logarithmic transformations were performed on variables with a skewness of >1. Data analysis was performed using SPSS version 20.0. Descriptive statistics were used to describe the characteristics of the sample as well as patient reported outcomes on quality of life, functional impairment, fatigue and mental health. Correlational analyses identified the main correlates of disease manifestation with different aspects of patient reported health status. RAND-36 score on physical functioning (RAND-36_PF), was used as indication for physical quality of life. Total scores were used for SIP, CIS and HADS as indication of functional impairment, subjective fatigue and mental health. Correlations lower than .30 were considered negligible, between .30 and .50 low, between .50 and .70 moderate and between .70 and .90 strong [19] (Hincle et al. 2003). Hierarchical multiple regressions were performed to explore the predictive value of genotype and clinical manifestation for patient reported outcomes. Genotype and clinical manifestation were first entered in the analyses, followed by patient reported outcomes. As dependent variables we focused on physical functioning, functional impairment, fatigue and mental health.

## Results

### Response

A total of 122 patients with the m.3243A > G mutation was invited to participate. Seventy-two patients completed the questionnaires, a response rate of 59 %. Demographic characteristics of the patients are summarized in Table 1. Fifty nine percent were women. Differences between response group and non response group could be assessed on demographic and clinical characteristics. Heteroplasmy levels did not differ between the two groups (t = −.705; *p* = .482). The same was true for NMDAS subscales 1 and 2. Non response group showed significantly more problems on the NMDAS 3 subscale (t = 6.771; *p* = .021) and nearly significant more problems on total NMDAS scale (t = 3.809; *p* = 0.053). Age (t = −.641; *p* = .523), BMI (t = −1.754; *p* = .082) and height (t = −1.208; *p* = .229) did not differ, however, non responders had a lower weight (t = −2.114; *p* = .036).

**Table 1:** General characteristics of patients (*n* = 72) as well as genotype and disease manifestation

	Mean	Median	Range
Age (years)	45	46	19–67
BMI (kg/m2)	24	23	14–41
Length (in cm)	168	167	142–193
Weight (in kg)	68	67	35–113
Heteroplasmy level (%)			
-leucocytes	19	13	0–56
-UEC	49	27	0–97
-buccal mucosa	34	18	0–73
NMDAS			
−1 current functioning	8	7	0–43
−2 system specific involvement	6	5	0–26
−3 current clinical assessment	3	4	0–29
total NMDAS	17	16	0–98

Response rate on repeated assessment at T2 and T3 was 50 (69 %) and 48 (67 %) respectively. Correlations on outcomes measures between T1 and T2 assessments varied from .837 (functional impairment) to .922 (mental health). Correlations between T1 and T3 assessments varied from .761 (functional impairment) to .858 (physical functioning).

### Genotype

Levels of heteroplasmy were assessed in Leucocytes (mean 19 %; SD = 13; range 0–56)), urinary epithelial cells (UEC; mean 49 %; SD = 27; range 0–97) and buccal mucosa (mean 34 %; SD = 18; range 0–73).

### Disease manifestation

Mean NMDAS score was 17 (SD = 15) varying from 1 to 98, median 14.5. Twenty-one percent of the patients showed mild symptoms, 50 % moderate and 29 % severe. There were no asymptomatic patients in the sample.

### Quality of life

Quality of life of patients with m.3243A > G mutation is presented in Table 2. Data show impairment in all quality of life domains. In comparison to age and sex matched norm groups, scores on all dimensions deviated from healthy people (t- values varied from −10,96 (General health: *p* < .001) to −2.98 (Mental health; *p* = .004)).

**Table 2:** Quality of life (RAND-SF36) scores compared to norms (*n* = 72)

	Mean^a^	SD	Norm group
Physical health	63.4	26.7	81.9
Social health	64.1	24.3	86.9
Physical role functioning	39.6	42.5	79.4
Emotional role functioning	69.4	40.2	84.1
Mental health	70.8	17.6	76.8
Vitality	49.0	18.8	67.4
Pain	68.3	25.9	79.5
General health	44.1	22.4	72.7
Experiences health change	43.1	21.1	52.4

^a^Higher scores reflect better quality of life

Scores on the RAND-36 subscale physical functioning indicated significantly more problems (mean 63,40) compared to norm group of comparable age and sex (mean 81.9; t-value −5.9; *p* < .001). Most limitations were experienced in putting great physical efforts, 61 % of the patients experienced serious impairments. About one third of the patients experienced serious impairments with climbing stairs and walking more than one kilometer. One out of five indicated serious problems with putting moderate physical efforts, carrying groceries or stooping. Only a one out of about ten patients or less indicated severe impairments with walking more than 500 m, climbing one stair or washing and dressing.

### Functional impairment

*Functional impairment* was assessed with the eight dimensions of the Sickness Impact Profile (SIP): Mean scores on home management were 86.89 (SD = 86.5; M = 54; range 0–328), on work 30.89 (SD = 59.1; M = 0; range 0–265), and on recreation 76.54 (SD = 71.7; M = 51; range 0–261) reflecting more functional impairments compared to healthy controls (t-values vary from 2.184; *p* = .032 (work) to recreation t = 8.016; *p* < .001).

74 % of the patients experienced no impairment in mobility, 57 % no impairment in ambulation. Mean mobility score was 39.5 (SD = 93; M = 0; range 0–2173), mean ambulation score was 60.0 (SD = 91; M = 0; range 0–494). Patients show more impairments than healthy comparison group (mobility t = 3.018; *p* = .004; ambulation t = 5.405; *p* < .001). Thirty-nine percent % of the patients experienced no impairments in alertness. Mean alertness score was 127,10 (SD = 157; M = 75; range 0–664), this was more than healthy controls (t = 16.36; *p* < .001).

### Fatigue

The majority of the patients with a m.3243A > G mutation reported abnormal levels of fatigue (78 %), 60 % reported severe levels of fatigue (*n* = 43, 60 %). The mean CIS fatigue score was 37.4 (SD 12.8, Table 3). Mean CIS_fatigue score of healthy controls was 17.3 (SD = 10.1). Patients show a little bit less fatigue than patients with MS (t = −1.832; *p* = .071) but less than patients with chronic fatigue syndrome (t = −9.439; *p* < .001). Age and gender did not differ between fatigued patients and non-fatigued patients.

**Table 3:** Fatigue as assessed by CIS compared to norms

	Mean	SD	Mean norms	SD	Patients vs norms
Perceived fatigue	37.4	12.8	17.3	10.1	t = 13.541**
Concentration	18.7	9.2	9.5	5.9	t = 8.525**
Motivation	14.8	6.3	7.9	4.1	t = 9.312**
Activity	11.9	6.0	6.6	4.5	t = 7.475**
total	82.8	30.6	41.5	19.1	t = 11.441**

** = *p* < .001

### Mental functioning

Mental functioning was assessed with the HADS. Results indicated that 26,4 and 31,6 % of the patients indicated clinical relevant symptoms of respectively depression and anxiety. 36,9 % scored above the cutoff of clinical relevant levels of general distress. Comparing the patient groups with healthy Dutch controls shows higher levels of depression (t = 3,092; *p* = .003) as well as total symptom scores in the patient group (t = 2,048; *p* = 0.44). Scores on anxiety were comparable to healthy control and better than a comparison group of general medical patients (t = −2,378; *p* = .020) [18].

### Relationship of patient reported outcomes with disease manifestation (NMDAS)

To assess to what extend patient reported outcomes were related to disease manifestation as indicated by NMDAS, ANOVA’s were performed with three NMDAS categories as factor and set of patient reported outcomes as dependent variables. ANOVA’s indicated that scores on physical functioning, functional impairment, fatigue and mental health differed significantly between NMDAS severity groups, with patients with worst clinical manifestation indicating most physical problems, most functional impairment, most fatigue and most mental health problems (Table 4).

**Table 4:** Patient reported outcomes by NMDAS categories mild (1–5), moderate (6–20), severe (>20)

	F value^a^
Physical functioning	25.364**
Functional impairment	19.568**
Fatigue	25.364**
Mental functioning	5.701*

^a^Statistic of ANOVA; ** *p* < .001; * *p* < .05

To assess the strength of the relationship between disease presentation and patient reported outcomes, spearman correlations between NMDAS subscale scores and different patient reported outcomes were calculated. Correlation of physical functioning, functional impairment, fatigue, and mental health with three subscales and total scale on NMDAS varies from strong (r > =.70) to negligible (r < .30). Perceived physical functioning was most strongly related with NMDAS scores with strong correlations with the NMDAS 1 (rho = −.727) and total score (rho = −.714), and moderate correlation with NMDAS 2 (rho = −.528) and 3 (rho = −.574). Functional impairment was most strongly related to NMDAS1 (rho = .665) and NMDAS3 (rho = .382). Fatigue is moderately correlated with NMDAS 1 (rho = .564) and total NMDAS (rho = .546), and weak with NMDAS 2 (rho = .446) and 3 (rho = .344). Mental health was only weakly correlated with NMDAS 1 (rho = .376), 2 (rho = .454) and total score (rho = .425). The correlation between mental health and NMDAS 3 was negligible.

### Prediction of physical functioning, participation, fatigue and mental functioning

To explore which factors predicted patient perceived health status, four aspects of perceived health were taken into account as outcomes in different regression analyses: physical functioning, impairments in daily functioning, fatigue and mental health. Hierarchical regression analyses were performed in which mitochondrial genetics and clinical manifestation were entered in the first steps, and subsequently physical functioning, functional impairment, fatigue and mental health in the next steps. The correlations are presented in Table  5.

**Table 5:** Correlations (spearmans’s rho) between genotype, clinical presentation and patient reported functioning on different domains N=72

	Physical functioning (RAND-SF36_PF)	Functional impairment (SIP total)	Fatigue (CIS perceived fatigue)	Mental health (HADS total)
Genotype	-.215	.178	.271*	.225*
NMDAS total score	-.714**	.558**	.546**	.425**
Physical functioning		-.695**	-.663**	-.504**
Functional impairment	-.695**		.743**	.669**
Fatigue	-.663**	.743**		.692**
Mental health	-.504**	.415**	.692**	

** p<.001; * p<.05

A first hierarchical multiple regression analysis was performed to predict physical functioning. Next to genotype, clinical manifestation was entered as predictor. The total model significantly predicted physical functioning (F (2,65) = 28.174; *p* < .001; R^2^ = 0.468). Heteroplasmy did not significantly explained variance in physical function, after controlling for genotype, NMDAS significantly added 45.0 % explained variance. In the total model, NMDAS (Beta = −.671; *p* < .001) was a significant predictor of physical functioning.

A second hierarchical multiple regression analysis was performed to predict functional impairment. Next to genotype and clinical manifestation, physical functioning was entered as predictor. The total model significantly predicted functional impairment (F (3,64) = 22.617; *p* < .001; R^2^ = 0.519). NMDAS (33,1 %) and physical functioning (15.9 %), significantly added explained variance. In the total model, only physical functioning (Beta = −.561; *p* < .001 was a significant predictors of functional impairment.

A third hierarchical linear regression was calculated to predict perceived fatigue based on genotype, clinical functioning, physical functioning, and functional impairment. A significant regression equation of the total model was found (F(4,63) = 29.461, *p* < .001), with an R^2^ of .655. Genotype explained 7.3 % variance, after controlling for genotype, NMDAS added another 9.0 %; physical functioning another 32.7 %, and, after controlling for previous factors, functional impairment added another 16.5 % of the variance.

In the total model, heteroplasmy (Beta = .586; *p* = .053), NMDAS (Beta = −.337; *p* = .002), physical functioning (Beta = −.445; *p* < .001), and functional impairment (Beta = .586; *p* < .001) significantly predicted perceived fatigue.

A last hierarchical linear regression analysis was calculated to predict mental health, also based on genotype, clinical manifestation, physical functioning, functional impairment, and fatigue. A significant regression equation of the total model was found (F (5,62) = 13.250; *p* < .001), with an R^2^ of .531. Heteroplasmy explained 6 % of the variance. After controlling for heteroplasmy, NMDAS additionally explained 9.1 % of the variance, physical functioning also 11.6 %, functional impairment 19.6 % and fatigue 5.8 %. In the total model, functional impairment (Beta = .396; *p* = .013) and fatigue (Beta = .412; *p* = .008) significantly predicted mental health.

## Discussion

Patients with the m.3243A > G mutation, experience important impairments in several domains of their health. Sixty percent showed severe levels of fatigue, and 37 % percent showed clinical relevant mental health problems. The variation in quality of life, functional impairment, fatigue and mental health could not be explained by level of heteroplasmy in body fluids and only partly by disease manifestation as indicated by NMDAS. This means that patients with a comparable clinical presentation can have a substantial variation in the impact of the disease in their daily life. In order to tailor treatment to complaints experienced by patients, integrating the perspective of the atient in clinical assessment is warranted [19] (Hymans 2011). This is especially important in mitochondrial diseases because of the chronic and disabling character of the disease and because of the focus on care rather than cure [20] (Wolters et al. 2013).

One of the explanations for the variation in patient reported health could be related to specific behaviour in response to the disease. For instance, being engaged in daily activities positively effects physical condition by being active, stimulates social functioning by being in contact with other people and stimulates mental health by increasing the possibility to experience positive events (see e.g. Apabhai et al. 2011 [21]). From studies in patients with other chronic medical conditions, such as Parkinsons Disease [22] (Simpson et al. 2014), Multiple Sclerosis [23, 24] (Trojan et al. 2007), Diabetes [7] (Goedendorp et al. 2014), Ehlors Danlos [9] (Voermans et al. 2010), Rheumatoid Artritis [26], COPD [27, 28] (Vercoulen et al. 2008); and cancer [15] (Servaes et al. 2002), we know that psychological factors could play a role in the explanation of these illness behaviours. Concerns about impact of activities on physical functioning or specific attributions regarding causes of complaints have been shown to mediate relationship between disease characteristics and daily functioning [29] (Lukkahatai et al. 2013). This could provide hope for patients as tailored care to support patients in management of daily activities could support their participation and so, indirectly, improve their daily functioning. However, future studies are needed to understand the specific role of these factors in patients with mitochondrial diseases in general and with m.3243A > G mutation more specifically.

This study included a broad range of patient reported outcomes based on standardised measures also shown to be sensitive for investigating these outcomes in other patient groups. In addition, also genotype and disease manifestation were taken into account reflecting the broad range of disease manifestation from genotype, to clinical phenotype, to daily functioning and perceived health. Quality of life and patient reported outcomes are only sporadically integrated in studies concerning patients with mitochondrial diseases. Mitochondrial diseases are characterised by a great variability in expression. This even more supports the need for systematic assessment of patient reported outcomes. This study focused on a relatively homogeneous group of patients carrying the same mutation. Including also patients with different diagnoses even increase this variability. This makes it even more important to built agreement on the conceptualisation and subsequent assessment of patient reported outcomes in patients with mitochondrial disease. Especially in small patient groups such as patients with the m.3243A > G mutation, agreement in outcome assessments could importantly increase gain in knowledge on variability in patient reported outcomes, and, even more important, its possible predictors.

This study also revealed interesting information regarding outcomes of clinical trials predominantly focusing on medical outcomes. This study shows that, next to survival rates and clinical manifestation, also patient perceived functioning on different health domains is important to explain the relevance of trial outcomes for patients’ daily functioning. It could also explain the possible role of psychological and behavioral mediators explaining the relationship between disease manifestation and perceived functioning [30, 31, 32] (Miller et al. 2010; Wolters et al. 2013, Zeltner et al. 2014).

Longitudinal research should shed more light on this issue. It could explain the role of these mediators in changes in perceived health. It could be highly assumed, for instance, that coping and cognitions regarding somatic complaints are important mediators able to explain variation in the relationship between disease activity and patients’ complaints. Future studies should also include more objective assessment of daily functioning e.g. exercise tests and by using registrations of daily movements. However, previous studies revealed that ambulation subscale of the SIP, as used in the present study, highly correlates with physical fitness and objective physical activity [16] (Vercoulen et al. 1999).

Another limitation is that absence of assessment of nutrition. Especially because of the relatively low weight of patients in the sample, low BMI could explain fatigue and poor physical functioning. Future studies should take nutrition and its effects on different domains of quality of life into account [33] (Arrom et al. 2014).

Future research has to further elaborate the development and efficient use of patient reported outcomes in the practice of mitochondrial medicine, as specific tools need to be validated for specific patient groups. Differentiation has to be made between instruments assessing general quality of life, disease specific complaints and specific bodily symptoms [20,32,34] (Wolters et al. 2013; Zeltner et al. 2014; Bann et al. 2015). Instruments assessing general quality of life are needed to compare outcomes of patients with mitochondrial disorders with patients with other, more or less comparable medical conditions. Disease specific measures could be used to compare the impact of the disease of a specific patient in relation to the whole patient group. These measures are also useful as outcomes for clinical trials because of their frequently reported higher sensitivity for chance.

Developing these measures for the field of mitochondrial medicine asks for integrating instruments available in other patient groups with information from interviews with patients to be able to point to specific quality of life aspects for patients with a mitochondrial disease. One could think about the specific burden of the inherited aspect of the disease, the lack of tangible symptoms of the disease in communicating about impairments in the social environment, the unpredictable course, the possible lethal character and the lack of control patients could have on the symptoms they experience. These specific characteristics need to be integrated in a mitochondrial disease specific instrument to assess quality of life, or burden of the disease.

## Conclusions

This study showed that patients reported considerable impairments in their quality of life and daily functioning. In addition, it indicated the complexity of the relationship between patient reported outcomes, clinical manifestation as well as genotype in patients with m.3243A > G mutation. This point to the importance of integrating patient reported outcomes in clinical assessment as well as clinical trials to gain more understanding in the complexity of factors explaining outcomes in these patients, as well as to provide starting points for interventions to support patients in the management of their disease.

## Abbreviations

ANOVA: analysis of variance
BMI: body mass index
CIS: checklist individual strength
COPD: chronic obstructive pulmonary disease
HADS: hospital anxiety and depression scale
NCMD: Nijmegen Center of Mitochondrial Disorders
MELAS: mitochondrial encephalomyopathy lactic acidosis and stroke like episodes
MIDD: maternally inherited diabetes and deafness
NMDAS: Newcastle Mitochondrial Diseases Adult Scale
SD: standard deviation
SIP: sickness impact profile
T1DM: type 1 diabetes mellitus
